# Determination of 27 bovine plasma amino acids and metabolites using zwitterionic-hydrophilic interaction liquid chromatography coupled with isotope dilution electrospray ionization triple quadrupole liquid chromatography-mass spectrometry and the effect of deproteinization timing

**DOI:** 10.3168/jdsc.2023-0449

**Published:** 2023-11-17

**Authors:** A.F. Ortega, M.E. Van Amburgh

**Affiliations:** Department of Animal Science, Cornell University, Ithaca, NY 14853

## Abstract

The use of zwitterionic-hydrophilic interaction liquid chromatography (Z-HILIC) columns for analysis of underivatized analytes has allowed simpler sample preparation of bovine plasma for sensitive and selective analysis, when coupled with mass spectrometry (MS). The objective of this study was to evaluate and validate this analytical technique to measure AA and metabolites in bovine plasma at 2 deproteinization times. A robust method using Z-HILIC coupled to a triple quadrupole MS (TQMS) was evaluated and validated to quantitatively analyze 19 AA using isotope dilution and 8 AA metabolites qualitatively in bovine deproteinized plasma. The timing of deproteinization was investigated to determine if plasma should be deproteinized upon collection (on-site) or immediately before analysis (in-lab). Analytes were separated using a Z-HILIC column in a 21 min run and analyzed with a TQMS in positive electrospray ionization for identification and quantification. The method was validated for standard curve linearity, limits of detection (LOD) and quantification (LOQ), intra- and interday precision (% coefficient of variation; CV), recovery (%), and freeze-thaw stability (% CV) after 1 mo. Coefficients of determination (R^2^) were over 0.993, and LOD and LOQ were below measured values for all AA. The CV for the intraday and interday precision were below 18%, except for cystine (Cys2) and Orn in-lab. Recoveries on-site and in-lab ranged from 75% to 120% for all analytes except Cys2 in-lab. Most analytes were stable after 1 mo of freezing regardless of deproteinization timing, CV <25%, except for hydroxyproline (Hyp). The concentration of Cys2 was affected by deproteinization in-lab compared with on-site, and even though Glu and Hyp were different between the 2 deproteinization timings, the concentrations between the 2 timings were within the standard deviation.

## Highlights

• We evaluated and validated a precise, accurate, and stable method for analysis of bovine AA and AA metabolites.
• We employed a novel approach using Z-HILIC coupled to a mass spectrometer.
• Cystine recovery is affected if plasma is not deproteinized immediately.
• Hydroxyproline is not stable after a 1-month freeze-thaw cycle.

## Graphical Abstract

**Figure:**
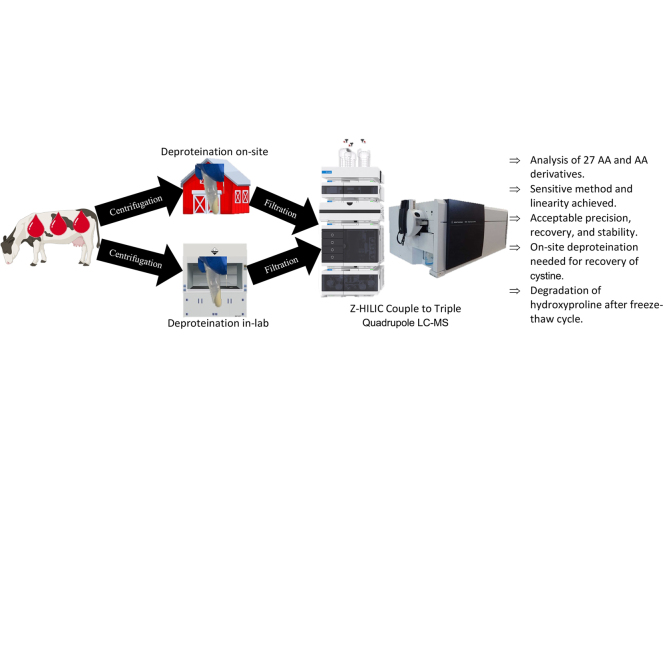
**Summary** This study evaluated and validated a zwitterionic-hydrophilic interaction liquid chromatography (Z-HILIC) and isotope dilution electrospray ionization triple quadrupole liquid chromatography-mass spectrometry method for analyzing amino acids (AA) and their derivatives in bovine plasma. The method demonstrated good quantitation results for linearity, precision, accuracy, and stability for most analytes, and the measured concentrations were consistent with published values. However, accurate recovery of cystine requires immediate deproteinization upon collection, and hydroxyproline was found to be unstable after a one-month freeze-thaw cycle. Overall, the method shows promise as a low-cost and efficient means of routinely analyzing plasma metabolites.

Amino acids and their derivatives play crucial roles in metabolic pathways, gene expression, immunity, oxidative defense, secretagogues, protein turnover, and cell signaling and physiology within the body (Wu, 2022). Therefore, the analysis of AA and related metabolites can provide valuable insights into the nutritional, metabolic, and health status of dairy cows. The traditional AOAC method (Toledo et al., 2021; Chandler et al., 2022; AOAC International, 2023) for analyzing AA uses ion exchange chromatography with postcolumn ninhydrin derivatization and norleucine as an internal standard. This method has been generally accepted for its reliability and sensitivity but can be time consuming, requires extensive sample preparation, and can suffer from reagent instability and interference (Dietzen et al., 2008; Rutherfurd and Gilani, 2009; Rigas, 2012). Additionally, the use of a single internal standard might not account for losses of all AA due to their different physio-chemical properties (Calder et al., 1999; Rutherfurd and Gilani, 2009). Moreover, isotope dilution (**ID**) GC-MS has been used to analyze tert-butyldimethylsilyl (**tDBMS**) derivatives of AA (Calder et al., 1999) and uses an isotopically labeled internal standard of all AA and improves analysis time and specificity. Yet, this method still requires thorough sample preparation and derivatization, and it has lower retention of polar analytes such as arginine (Peace and Gilani, 2005; Kaspar et al., 2009; Krumpochova et al., 2015). Recently, Toledo et al. (2021) validated a method for quantifying underivatized AA using normal phase ion exchange chromatography with single quadrupole MS. This technique showed promising results but was limited in the number of analytes. An alternative method that has started to become more widely available is hydrophilic interaction liquid chromatography (**HILIC**; Alpert, 1990). HILIC offers an alternative approach that uses a polar column for separation by using a gradient with a highly organic mobile phase and water as the eluting solvent. This technique has become a popular analytical tool because it does not require derivatization, resolves isobaric compounds, provides stronger retention of polar compounds, improves ionization for better sensitivity, and can be coupled with MS for increased selectivity (Petritis et al., 2002; Jandera, 2011; Buszewski and Noga, 2012; Krumpochova et al., 2015). Various phases of HILIC have been developed, such as **Z-HILIC**, which contains a zwitterionic ligand that improves ion exchange capacity (Kambhampati et al., 2019; Lioupi et al., 2022).

Therefore, the aim of this study was to validate a method using ID Z-HILIC and electrospray ionization (**ESI**) triple quadrupole MS (**TQMS**) for analysis of AA and AA metabolites in deproteinized bovine plasma. Further, this study investigated the effect of deproteinization timing by deproteinizing plasma immediately after collection at the farm (on-site) or in the laboratory before analysis (in-lab). The method was validated at both timings for calibration curve linearity, limits of detection (**LOD**) and quantification (**LOQ**), recovery, interday and intraday precision, freeze-thaw stability after 1 mo.

Acetonitrile, methanol (MeOH), and water were purchased from Avantor, 99% formic acid (**FA**) from Thermo Scientific, 5M ammonium formate (**AF**) from Agilent (California), and all were liquid chromatography (**LC**)-MS grade. Seraprep was purchased from Pickering Laboratories (California). All reference AA and standard AA mixes (A6282 and A6407) were purchased from Sigma-Aldrich (Missouri), and a mix of U-^13^C,^15^N labeled canonical AA (MSK-CAA-1), used as an internal standard, was purchased from Cambridge Isotope Laboratories (Massachusetts). A mixed standard stock was prepared by combining the 2 standard AA mixes with Gln, Gly, and urea dissolved in 0.1 *N* HCl. The concentrations for the AA and metabolites analyzed were as follows: urea, 123.58 μg/mL; creatinine, 8.96 μg/mL; Phe, 29.44 μg/mL; Leu, 23.38 μg/mL; Ile, 23.38 μg/mL; Trp, 16.17 μg/mL; Met, 26.59 μg/mL; Val, 20.89 μg/mL; Pro, 20.57 μg/mL; Tyr, 32.29 μg/mL; hydroxyproline (**Hyp**), 23.36 μg/mL; Ala, 15.88 μg/mL; Thr, 21.23 μg/mL; Gly, 29.22 μg/mL; Gln, 69.53 μg/mL; Asn, 23.54 μg/mL; Ser, 18.73 μg/mL; Cit, 31.22 μg/mL; 1-methyl-histidine (**1MH**), 13.40 μg/mL; Glu, 26.22 μg/mL; His, 12.29 μg/mL; 3-methyl-histidine (**3MH**), 13.40 μg/mL; carnosine, 17.92 μg/mL; Arg, 13.80 μg/mL; cystine (**Cys2**), 21.41 μg/mL; and Orn, 10.47 μg/mL. Working standards were prepared from the stock by dilution using the initial mobile phase to the appropriate concentrations of the calibration curve.

The Cornell University Institutional Animal Care and Use Committee approved all animal procedures (protocol 2021–0064). Plasma was collected from Holstein dairy cows using sodium heparin tubes and separated by centrifugation. To investigate the effect of deproteinization timing an aliquot of plasma was deproteinized on-site and another aliquot was frozen and deproteinized in-lab. For deproteinization, equal volumes of plasma and Seraprep were added, vortexed, and placed on ice for 16 h at 4°C. Samples were then centrifuged at 15,700 × *g* for 10 min at 4°C, the supernatant was diluted 1:10 using solvent B (described below) and filtered through a 2-μm syringe filter.

The analysis was performed using an Agilent 1260 Infinity II LC system consisting of a multisampler, a binary pump, and a multicolumn thermostat (Agilent). Separation of extracted analytes was performed on an Atlantis Premier ethylene-bridged hybrid (BEH) Z-HILIC column (2.1 × 150 mm, 2.5 μm; Waters, MA) equipped with an Atlantis Premier BEH Z-HILIC 2.5 μm VanGuard cartridge precolumn (Waters). Solvent A was 0.1% FA + 10 m*M* AF in water and solvent B was 0.1% FA + 10 m*M* AF in 90% acetonitrile. A step gradient was used as follows: 0% A from 0 to 5 min, 20% A from 5 to 6 min, 30% A from 6 to 7 min, 50% A from 7 to 8 min, and 80% A from 8 to 13 min. The column was equilibrated using 0% A from 13 to 21 min. The flow rate was 0.4 mL/min at a column temperature of 40°C. A multiwash cycle was performed between injections on the needle and seat back as follows: 30 s at 50% methanol, 30 s at 10% acetonitrile, and 60 s at 90% acetonitrile. The multisampler injected 1 μL of the stable isotope-labeled internal standard mix and 1 μL of the prepared sample, and the column eluent was directed to the MS. The LC system was coupled to an Agilent 6460 TQMS in positive ESI using Agilent Jet Stream ESI source. The optimized source parameters were capillary voltage of 2,000 V, source temperature of 290°C, source flow of 11 L/min, sheath gas temperature of 350°C, sheath gas flow of 12 L/min, and nebulizer N gas pressure of 310.3 kPa. The ion transitions, fragmentor voltage, and collision energies were optimized for all analytes using the optimizer tool of the MassHunter software (version 10.1, Agilent), and analytes were detected by dynamic multiple reaction monitoring (Table 1). At least 2 product ions were chosen for each analyte and the most abundant ion was used for quantification. Instrument control, data acquisition, and qualitative and quantitative data analysis was done using MassHunter workstation software (Agilent).

**Table 1:** Triple quadrupole liquid chromatography-MS parameters for dynamic multiple reaction monitoring and sensitivity (limit of detection [LOD], limit of quantification [LOQ]) of AA and metabolites analyzed

Analyte	Retention time (min)	Precursor ion (*m*/*z*)	Quantitative product ion (*m*/*z*)	Qualitative product ion (*m*/*z*)	Fragmentor voltage (V)	Collision energy (eV) quantitative product ion	Collision energy (eV) qualitative product ion	LOD (ng/mL)	LOQ (ng/mL)
1, Urea	2.8	61.0	44.1	44.7	48	24	20	74.06	246.86
2, Creatinine	3.1	114.1	44.2	86.1	92	16	8	54.15	180.51
3, Phe	4.9	166.1	120.1	103.1	72	12	30	0.51	1.70
4, Leu	5.2	132.1	86.1	44.1	74	8	28	4.63	15.44
5, Ile	5.5	132.1	86.1	69.1	72	8	16	2.84	9.46
6, Trp	5.6	205.1	187.9	146.0	70	4	15	0.99	3.31
7, Met	6.0	150.1	56.1	104.0	70	16	8	0.44	1.48
8, Val	6.5	118.1	72.1	55.1	70	8	22	2.60	8.66
9, Pro	6.7	116.1	70.1	43.1	70	16	29	0.81	2.70
10, Tyr	6.9	182.1	91.1	136.1	72	30	12	5.09	16.96
11, Hyp^1^	8.0	132.1	86.1	68.1	78	12	20	1.81	6.03
12, Ala	8.1	90.1	44.1	45.1	48	8	40	10.39	34.63
13, Thr	8.2	120.1	56.1	74.1	72	16	4	6.99	23.30
14, Gly	8.7	76.0	30.1	48.0	48	4	4	24.58	81.93
15, Gln	8.8	147.1	130.0	84.1	72	8	16	30.12	100.41
16, Asn	9.0	133.1	74.1	87.1	72	12	8	12.29	40.97
17, Ser	9.0	106.1	60.3	42.1	72	12	28	9.34	31.14
18, Cit	9.3	176.1	159.0	70.1	78	8	28	21.48	71.60
19, 1MH^1^	9.4	170.1	124.1	83.1	94	12	28	3.54	11.81
20, Glu	9.4	148.1	84.0	56.1	72	16	32	1.71	5.71
21, His	10.0	156.1	110.1	95.1	70	12	16	4.80	16.01
22, 3MH^1^	10.1	170.1	109.1	126.1	106	16	12	0.15	0.51
23, Carnosine	10.2	227.1	110.0	155.9	74	24	12	0.57	1.90
24, Arg	10.3	175.1	70.1	60.1	82	24	12	6.42	21.39
25, Lys	10.4	147.1	84.1	130.1	72	16	8	35.73	119.11
26, Cys2^1^	10.4	241.0	74.1	151.8	84	28	12	8.53	28.45
27, Orn	10.5	133.1	70.1	115.9	72	20	8	2.78	9.27

1 Hyp = hydroxyproline; 1MH = 1-methyl-histidine; 3MH = 3-methyl-histidine; Cys2 = cystine.

The method was validated following the guidelines from the International Conference on Harmonization (ICH, 1996) for each of the deproteinization timings. For evaluation of linearity, at least 5 concentrations were selected for the standard curve analysis of each analyte. For quantitative analysis of 19 proteogenic AA the linearity was determined by using an isotopically labeled internal calibration standard with or without a 1/× weighting factor. For the qualitative analysis of the remaining analytes, an external calibration was used with or without a 1/× weighting factor. Calculated standard values were accepted within ±20% of the theoretical value. The coefficient of determination (R^2^) had to be ≥0.990. The LOD were determined at a signal-to-noise ratio (**S/N**) of 3.3 and LOQ at a S/N of 10, and the LOD and LOQ were calculated from at least 10 blanks by multiplying 3.3 or 10, respectively, by the standard deviation of the average blank response (σ) divided by the slope of the standard curve (s; LOD = 3.3σ/s; LOQ = 10 σ/s). The LOD and LOQ are summarized in Table 1. Repeatability precision was measured as the % CV of 6 replicates freshly prepared within a day. Interday precision was calculated by analyzing triplicates for 3 consecutive days and calculating the % CV. Precision CV values had to be ±20% for acceptance. The recovery test was performed by adding an AA mix solution containing all analytes at least 0.5× their concentration before extraction. Four nonspiked samples and 4 spiked samples were run simultaneously, and percent recovery was estimated by subtracting the spiked amount by the amount observed divided by the amount spiked. Accepted recoveries had to be within ±25%. The 4 nonspiked samples from the accuracy run were frozen at −20°C and thawed after 1 mo for stability analysis by calculating the % CV. The ionization matrix effect was monitored by calculating the sample internal standard recovery from the average internal standard response of the standard curve, which had to be within ±25%. The method was further applied using both deproteinization timings to analyze AA and metabolites in samples that were collected as part of an unrelated experiment and had been stored at −80°C. To compare the timing of deproteinization, the plasma AA or metabolite concentration (**p[AA/M]**) measured for the precision (n = 12), accuracy (n = 4), stability (n = 4), and application (n = 9) runs were averaged for each timing and compared with a 2-sample paired *t*-test using the “t_test” function of the RSTATIX package (Kassambara, 2023) in R version 4.2.0 (R Core Team, 2022). Significance was declared at *P* < 0.01.

Linearity of the external standard curve was determined by using linear calibration, except for urea, all excluded the origin, and the weighting factor was determined for each analyte to improve recovery. For urea, a quadratic relationship was used for calibration to improve accuracy and allow a wider range of concentrations to be used. A 1/× weighting factor was used for the calibration curve of urea, Met, Tyr, Asn, 1MH, 3MH, carnosine, Arg, Lys, Cys2, and Orn, whereas the remainder of the analytes did not have any weighting factors. The R^2^ values ranged from 0.9933 (Cys2) and 0.9937 (Cit) to 0.9998 (urea) and 0.9996 (Asn, Lys), averaging 0.9975 for all analytes. As observed in Table 1, the method has outstanding sensitivity with the LOD ranging from 0.15 ng/mL for 3MH to 74.06 ng/mL for urea, and the LOQ ranging from 0.51 to 246.86 ng/mL for the same metabolites. The lowest point in the generated standard curves for each analyte was always greater than their respective LOQ. The use of an isotopically labeled internal standard normalizes the standard curve for the 19 proteogenic AA. Notwithstanding, the metabolites analyzed without an internal standard yielded sensitive results.

Precision, sample recoveries, freeze-thaw stability, and p[AA/M] results for both approaches of deproteinization are in Table 2. The intraday and interday CV were below 17.58% for all analytes except Orn and Cys2 and only when plasma was deproteinized in-lab. The intraday and interday precision for Orn was 21.98% and 20.43% CV, respectively, and for Cys2 it was 35.33% and 35.05% CV, respectively. The higher CV for Orn can explained by the low resolution of this metabolite and not having an isotopically labeled internal standard. Alternatively, the high CV of Cys2 can be explained by the low recovery of this AA when plasma is deproteinized in-lab. For both deproteinization timings, the recovery of AA and metabolites was between 75.3% and 119.8% for Ser and Cit on-site, respectively, except for Cys2 in-lab. Only 55.3% of Cys2 was recovered when plasma was deproteinized in-lab, which is consistent with findings from others due to Cys2 binding to plasma proteins (Schaefer et al., 1987; Fekkes, 1996). As seen in Figure 1, the magnitude of the peak of in-lab Cys2 is comparable to the noise surrounding it, whereas for on-site Cys2 the peak is at least 10 times larger than noise for appropriate quantification. Moreover, most AA and metabolites were stable after 1 mo of freezing with CV lower than 20.22% except for 3MH in-lab and Hyp both on-site and in-lab. Hydroxyproline varied from the original samples by 27.78% and 30.12% on-site and in-lab, respectively, showing a consistent degradation from the freeze-thaw cycle. The CV for 3MH when plasma was deproteinized in-lab was 25.37%; on the contrary the CV on-site was 17.75%, which was below the acceptance criteria of 20%. Last, as expected the p[AA] of Cys2 was significantly different (*P* < 0.001) for the 2 deproteinization timings. Further, Glu (*P* = 0.007) and Hyp (*P* = 0.006) were also different between on-site and in-lab deproteinization, but the difference between the 2 deproteinization timings were within the standard deviation. Overall, the p[AA/M] agreed with published values. Concentrations of the proteogenic AA and Cit were all within the ranges of recent publications (Patton et al., 2015; Martineau et al., 2017). The concentration of Orn in this study was 20.6 μ*M* on-site and 19.8 μ*M* in-lab, which are not within the range provided by Martineau et al. (2017) of 30 to 82 μ*M*, although Chandler et al. (2022) showed concentrations as low as 21.7 μ*M*. Concentrations of the other metabolites (urea, creatinine, 1MH, 3MH, carnosine) were also within the ranges found in the literature (Fetter et al., 2021; Premi et al., 2021; Chandler et al., 2022), except for Hyp for which the present study had higher concentrations. Since Cys2 is bound to plasma proteins, the concentrations presented in this paper only reflect free Cys2 and not total Cys2. For analysis of total Cys2, dithiothreitol must be used to free Cys2 from proteins.

**Table 2:** Intra- and interday precision, sample recoveries, freeze-thaw stability after 1 mo, and plasma AA or metabolite concentration (p[AA/M]) of all analytes at 2 timings of deproteinization

Analyte	On-site							In-lab						
	Precision (% CV)		Recovery		Stability	p[AA/M]		Precision (% CV)		Recovery		Stability	p[AA/M]	
	Intraday	Interday	%	% CV	% CV	ng/mL	μ*M* ± SD	Intraday	Interday	%	% CV	% CV	ng/mL	μ*M* ± SD
1, Urea^1^	5.11	13.13	94.4	4.66	13.70	231,377.13	3.85 ± 0.52^1^	3.68	10.27	105.3	2.75	5.72	245,746.84	4.09 ± 0.69^1^
2, Creatinine	2.45	10.99	85.6	4.29	1.31	8,081.07	71.44 ± 19.51	2.31	5.61	87.1	2.48	8.00	8,435.81	74.58 ± 20.09
3, Phe	1.87	14.86	100.1	5.88	2.99	7,335.76	44.41 ± 4.84	1.86	7.41	109.7	2.04	10.44	7,619.72	46.13 ± 4.84
4, Leu	1.67	14.97	99.3	5.88	0.14	17,330.71	132.13 ± 33.86	3.04	6.80	108.8	2.08	5.70	18,111.17	138.08 ± 37.27
5, Ile	1.31	14.74	104.1	7.32	2.07	12,862.66	98.07 ± 14.46	2.37	8.79	112.4	3.18	2.87	13,341.17	101.71 ± 16.24
6, Trp	4.30	17.92	101.7	7.50	2.64	5,183.36	25.38 ± 7.39	1.92	10.00	114.8	2.07	3.05	5,440.48	26.64 ± 7.72
7, Met	2.68	14.63	103.5	5.84	0.24	4,205.93	28.19 ± 4.95	7.84	9.18	119.8	2.59	7.26	4,180.27	28.02 ± 4.28
8, Val	3.24	10.54	98.6	3.82	0.57	23,666.26	202.02 ± 53.08	3.80	2.95	106.5	3.48	7.73	24,201.05	206.58 ± 57.25
9, Pro	5.36	11.54	93.2	4.33	2.70	6,989.20	60.71 ± 10.79	7.03	6.38	101.5	3.69	9.50	7,127.81	61.92 ± 10.99
10, Tyr	5.36	14.84	117.3	11.47	0.85	6,530.27	36.05 ± 8.24	11.70	11.60	116.2	3.77	8.46	6,752.41	37.27 ± 8.95
11, Hyp^2^	5.71	6.32	102.8	3.11	27.78	7,803.02	59.51 ± 11.96^a^	4.91	5.72	118.3	6.09	30.12	8,325.97	63.50 ± 15.03^b^
12, Ala	6.48	9.75	95.9	7.93	2.69	19,060.73	213.95 ± 31.90	8.65	5.46	100.0	7.19	10.56	19,159.27	215.05 ± 35.12
13, Thr	13.88	12.57	102.7	4.46	5.93	7,277.71	61.10 ± 18.06	11.19	15.05	101.0	8.83	20.22	7,347.15	61.68 ± 18.19
14, Gly	9.47	10.92	87.0	10.75	10.43	29,044.73	386.91 ± 120.66	14.81	11.47	76.3	11.74	10.85	30,993.02	412.86 ± 150.95
15, Gln	10.04	11.52	103.1	6.90	9.59	47,763.08	326.84 ± 168.72	13.62	17.03	94.0	6.32	5.28	48,087.95	329.06 ± 185.99
16, Asn	8.44	15.10	115.6	10.17	2.63	5,353.11	40.52 ± 12.19	8.91	15.32	97.8	11.16	10.62	5,960.46	45.12 ± 10.74
17, Ser	10.05	11.24	75.3	8.36	7.19	9,408.60	89.53 ± 11.08	10.62	12.63	102.5	7.83	4.82	9,239.41	87.92 ± 12.91
18, Cit	13.83	8.94	119.8	7.14	3.96	9,614.63	54.88 ± 8.22	14.21	8.85	94.2	7.00	16.47	9,753.73	55.68 ± 9.88
19, 1MH^2^	11.10	6.93	84.3	7.57	1.09	1,419.22	9.65 ± 2.72	11.37	6.97	100.2	8.96	16.12	1,372.13	9.33 ± 2.38
20, Glu	9.62	12.73	103.2	7.23	19.48	6,423.30	37.97 ± 9.35^a^	6.68	14.59	116.9	8.47	13.82	6,976.52	41.24 ± 11.39^b^
21, His	12.92	9.44	79.8	13.16	6.10	5,658.64	36.48 ± 5.80	10.44	9.39	88.4	8.05	8.12	5,322.42	34.31 ± 4.44
22, 3MH^2^	14.45	11.31	79.1	7.13	17.75	1,198.48	7.09 ± 1.19	17.17	10.03	79.8	6.38	25.37	1,174.29	6.95 ± 1.11
23, Carnosine	14.65	13.01	90.1	10.04	6.95	1919.48	8.49 ± 2.16	15.77	11.39	90.5	9.61	17.67	1,789.81	7.91 ± 1.19
24, Arg	13.91	10.87	92.8	10.88	0.20	9,290.61	53.34 ± 16.83	12.16	13.95	95.9	6.63	9.50	9,154.56	52.56 ± 16.92
25, Lys	17.58	13.26	82.3	12.71	4.00	7,725.85	52.85 ± 21.02	15.50	12.95	87.3	8.32	12.71	7,460.37	51.04 ± 19.28
26, Cys2^2^	13.75	11.55	82.4	14.86	5.63	2,854.76	11.88 ± 4.45^a^	35.33	35.05	55.8	14.64	1.49	780.25	3.25 ± 0.88^b^
27, Orn	14.30	12.63	103.8	11.08	10.89	2,723.95	20.62 ± 6.97	21.98	20.43	78.8	5.30	10.47	2,620.34	19.83 ± 7.57

a,b Different superscripts within a row signify a significant difference at *P* < 0.01.

1 Urea plasma concentration expressed in m*M*.

2 Hyp = hydroxyproline; 1MH = 1-methyl-histidine; 3MH = 3-methyl-histidine; Cys2 = cystine.

In conclusion, an efficient and robust method has been evaluated and validated using ID-Z-HILIC-ESI-TQMS for analyzing 27 AA and their derivatives in bovine plasma after on-site and in-lab deproteinization. The method demonstrated good quantification results for all analytes with linearity shown through R^2^ values over 0.993 and LOD ranging from 0.51 to 74.1 ng/mL and LOQ ranging from 1.7 to 246.9 ng/mL. The intraday and interday precision CV were below 22.0%, and recoveries ranged from 75.3% to 119.08%, except for Cys2 in-lab. Moreover, all analytes except for Hyp were found to be stable after being frozen for 1 mo. If Cys2 and Hyp are of interest, it is recommended to deproteinize the plasma immediately upon collection and thaw the plasma only once. Finally, the plasma concentrations of the analytes were consistent with published ranges for dairy cows. This analytical method provides convenient sample preparation without requiring derivatization or ion-pairing reagents, short run times, and low costs, making it suitable for the routine analysis of bovine AA and their derivatives.
